# The Impact of Arsenic, Cadmium, Lead, Mercury, and Thallium Exposure on the Cardiovascular System and Oxidative Mechanisms in Children

**DOI:** 10.3390/cimb47070483

**Published:** 2025-06-25

**Authors:** Marcin Wróblewski, Justyna Miłek, Antoni Godlewski, Joanna Wróblewska

**Affiliations:** 1 Department of Medical Biology and Biochemistry, Faculty of Medicine, Ludwik Rydygier Collegium Medicum in Bydgoszcz, Nicolaus Copernicus University in Toruń, 24 Karłowicza St., 85-092 Bydgoszcz, Poland; joanna.wroblewska@cm.umk.pl; 2 Department of Chemical and Bioprocess Engineering, Faculty of Chemical Technology and Engineering, Bydgoszcz University of Science and Technology, 3 Seminaryjna St., 85-326 Bydgoszcz, Poland; jmilek@pbs.edu.pl; 3 Student Scientific Club of Biochemistry and Bioorganic Chemistry, Department of Medical Biology and Biochemistry, Faculty of Medicine, Ludwik Rydygier Collegium Medicum in Bydgoszcz, Nicolaus Copernicus University in Toruń, 24 Karłowicza St., 85-092 Bydgoszcz, Poland; 319905@stud.umk.pl

**Keywords:** blood pressure regulation disorders, cardiovascular health, children, heavy metals, hypertension

## Abstract

Environmental exposure to heavy metals seriously threatens children’s health, potentially impacting the cardiovascular system. Mechanisms such as oxidative stress, inflammation, and lipid metabolism disturbances play a significant role in this process. Although cardiovascular diseases typically manifest in adulthood, an increasing number of studies suggest that their origins trace back to childhood and result from long-term pathophysiological changes. Therefore, early identification of modifiable risk factors is crucial for effective preventive measures and reducing future health risks.

## 1. Introduction

In recent decades, there has been growing interest in the impact of heavy metals on human health, particularly in the context of the pediatric population, which is especially vulnerable to the toxic effects of these elements due to rapid biological development and the immaturity of detoxification systems. Toxic metals such as lead (Pb), cadmium (Cd), mercury (Hg), thallium (Tl), and arsenic (As) have no physiological role in the human body. Their presence is associated with metabolic disturbances, oxidative stress, and damage to the cardiovascular system [1,2,3,4]. These compounds are widely present in the environment, and sources of exposure in children include not only food, air, and water but also everyday items such as toys and children’s jewelry [5,6,7]. Children are uniquely vulnerable to environmental exposure due to their frequent hand-to-mouth behavior, proximity to the ground during play, and higher intake of air, water, and food per kilogram of body weight compared to adults. Familiar sources include inhaling contaminated dust or air in industrial or urban areas, ingesting contaminated food such as rice, leafy vegetables, and fish high in methylmercury, and dermal absorption from items like cosmetics or plastic toys. Moreover, polluted drinking water, especially in regions with inadequate infrastructure or proximity to mining or industrial sites, is a major arsenic and lead exposure route. These environmental exposures are often compounded in low-income or high-risk areas, disproportionately burdening disadvantaged communities [4,6,7,8].

Exposure to heavy metals early in life may lead to persistent and adverse changes in the cardiovascular system, such as endothelial dysfunction, arterial wall thickening, reduced vascular elasticity, and structural alterations in the heart muscle [3,9]. Elevated levels of arsenic, lead, and cadmium in the body have been associated with disturbances in heart rhythm, increased blood pressure (BP), and impaired lipid profiles [2,10]. Due to their sensitivity and developing biological systems, young individuals are particularly vulnerable to the toxic effects of heavy metals. Research on their cardiovascular health impacts is growing, as subclinical vascular changes may increase the risk of heart disease in adulthood [11]. Cardiovascular disease (CVD) remains a growing public health issue worldwide, accounting for nearly one-third of all global deaths [11]. Long-term exposure to certain heavy metals has been associated with an increased risk of major adverse cardiovascular events, including acute myocardial infarction, heart failure, stroke, angina, atherosclerosis development, and cardiovascular death [12,13].

Early childhood exposure to heavy metals can affect the regulation of vascular tone by disrupting the production of nitric oxide (•NO), a key molecule essential for proper vascular function [14]. •NO, synthesized by nitric oxide synthases, plays a fundamental role in regulating vascular tone by inducing vasodilation. Maintaining adequate •NO bioavailability is crucial for preserving endothelial function and ensuring appropriate vascular responsiveness [15]. Under physiological conditions, redox homeostasis ensures the maintenance of proper concentrations of the •NO. However, oxidative stress, resulting from excessive production of reactive oxygen species (ROS), leads to endothelial cell damage, impairs vascular function, and reduces •NO bioavailability. Consequently, this contributes to developing endothelial dysfunction, arterial stiffness, and hypertension, which are early stages of atherosclerotic processes. As highlighted in the literature, oxidative stress plays a central role in the pathophysiology of hypertension, atherosclerosis, and CVD, leading to premature vascular aging, cardiac remodeling, and impaired heart function [15]. Heavy metals can affect human health through multiple mechanisms, including oxidative stress, inflammation, and endocrine disruption [13,16]. These toxicants interfere with the balance of key antioxidant enzymes such as superoxide dismutase (SOD), catalase (CAT), and glutathione peroxidase (GPx), thereby exacerbating oxidative stress and contributing to endothelial dysfunction and vascular impairment [13].

Studies have shown that heavy metals can influence inflammatory pathways, leading to elevated levels of inflammatory markers such as interleukin-6 (IL-6) and C-reactive protein (CRP), thereby placing additional strain on the cardiovascular system [14]. These processes can initiate chronic inflammation and lead to premature vascular aging in children, thereby increasing the risk of CVD later in life [13]. Inflammation and endocrine disruption may contribute to dyslipidemia and increased risk of CVD. Recent evidence suggests that early-life exposure to heavy metals is associated with altered lipid profiles in children, including elevated total and LDL cholesterol levels. These associations are hypothesized to be mediated by oxidative stress, inflammatory responses, and hormonal imbalances triggered by toxic metal exposure [16].

Prolonged exposure to heavy metals, especially during prenatal and early childhood, may induce lasting epigenetic changes that affect the expression of genes involved in inflammation and vascular tone regulation, ultimately increasing susceptibility to CVD later in life [14].

The selection of these five metals in this study is based on their well-documented toxicity, widespread presence in the environment, and growing or confirmed association with cardiovascular dysfunction, particularly in children. These metals are considered among the most hazardous environmental pollutants due to their ability to accumulate in biological tissues and their long biological half-lives. They remain a significant public health concern in developing countries [17,18,19]. Although thallium is less frequently discussed in population-based studies, its high toxicity and ability to disrupt potassium balance (crucial for cardiac electrical activity) make it an essential element in the toxicological analysis of the cardiovascular system [20,21]. These five metals represent both high environmental relevance and significant biological toxicity, providing a coherent and scientifically justified basis for assessing cardiovascular risk in children from heavy metal exposure.

This study aims to provide a comprehensive analysis of the toxic properties of heavy metals (mercury, lead, cadmium, arsenic, and thallium), with particular emphasis on their impact on the cardiovascular system in children. Based on current research, this literature review offers insights into sources of exposure, toxic mechanisms, biomarkers of exposure, and potential long-term health effects. Such knowledge is essential for developing effective preventive and intervention strategies to protect the most vulnerable population groups.

## 2. Mercury

Mercury is a widely occurring heavy metal that has no function in human metabolism. At the same time, the human body lacks adequate mechanisms for eliminating this element, which may lead to serious health consequences [22,23]. Mercury exists in three forms: elemental, organic, and inorganic. Inorganic mercury includes liquid metallic mercury and its vapors (Hg^0^), as well as monovalent (Hg^+^) and divalent (Hg^2+^) salts [22,23,24]. Mercury is most commonly found in the form of cinnabar (mercury sulfide, HgS), and its average concentration in the Earth’s crust is 0.09 mg/kg. Organic mercury, in which mercury atoms are bonded to carbon groups, includes methylmercury (CH₃Hg, MeHg), ethylmercury (C_2_H_5_Hg, EtHg), and phenylmercury (C_6_H_5_Hg, PhHg) [23,24].

The biological properties and toxicity of different forms of mercury depend on their chemical structure [4,23]. Elemental mercury readily crosses cell membranes and the blood–brain and placental barriers due to its high lipid solubility. It can also be transmitted to infants via breast milk, leading to its bioaccumulation in key organs such as the liver, brain, kidneys, and muscles, increasing the risk of toxic damage [23]. Once absorbed into the body, it is oxidized in red blood cells and tissues to inorganic forms (Hg^+^ and Hg^2+^) in the presence of CAT and peroxidase [23]. The divalent form (Hg^2+^) is more toxic than the monovalent form (Hg^+^) because it binds more easily to proteins and disrupts cellular function. In the body, the Hg^2+^ ion binds to sulfhydryl (-SH) groups of cysteine found in erythrocytes, glutathione (GSH), and metallothioneins, or is transported in plasma in suspended form [4,23]. Once bound, mercury disrupts redox homeostasis, impairs mitochondrial function, depletes GSH levels, and promotes the generation of ROS, ultimately increasing lipid peroxidation and contributing to cardiovascular and neurological toxicity. Mercury’s high affinity for selenium (Se) also reduces the activity of key antioxidant enzymes, including GPx, CAT, and SOD, thereby exacerbating oxidative stress and cellular dysfunction [23]. Methylmercury, considered the most toxic mercury, has a powerful affinity for -SH groups, leading to enzyme inactivation, cellular homeostasis disruption, and oxidative stress induction [24]. Mercury promotes excessive production of ROS, including •NO and nitrotyrosine, and enhances lipid peroxidation, contributing to oxidative stress and damage to macromolecules such as proteins and cell membranes. These disturbances may lead to endothelial dysfunction and increased vascular resistance, which can significantly affect the function of the cardiovascular system [23,25,26]. Moreover, mercury binds to metallothioneins, replacing zinc, copper, and other trace elements, and also competes with selenium, reducing the effectiveness of metalloenzymes [23,27]. The formation of mercury–selenium complexes reduces the availability of selenium for the synthesis of GPx, particularly GPx4, which plays a key role in protecting lipid membranes from peroxidation. The binding of methylmercury (MeHg) to the selenol group (-SeH) of GPx4 decreases its activity, leading to enhanced accumulation of ROS and promoting the process of ferroptosis [23,28]. Mercury can also influence platelet aggregation and coagulation processes, contributing to altered blood flow and increased BP [25].

Elemental mercury can be a source of exposure through inhalation of its vapors, which are released from broken thermostats during gold mining, as well as through the use of dental amalgam fillings, skin-lightening cosmetics, traditional medicines, and religious or cultural rituals such as voodoo. Inorganic mercury is found in some antiseptics, laxatives, cosmetics, and bactericidal, fungicidal, and insecticidal preparations. Organic mercury, in methylmercury and ethylmercury, is used in some pharmaceutical products, including vaccines preserved with thimerosal [25]. Additionally, specific fish species have high methylmercury concentrations due to their position in the food chain and long lifespan. Fish with high mercury levels include shark, swordfish, king mackerel, bluefin tuna, and marlin. In contrast, fish with low mercury levels, including salmon, cod, pollock, trout, sardines, and herring, are considered safer for consumption. Apart from fish, mercury may also be present in rice, particularly in areas with high levels of environmental contamination [29]. Regarding intake guidelines, the World Health Organization has set a provisional tolerable weekly intake for methylmercury at 1.6 μg per kilogram of body weight [29]. In urine, mercury levels above 10–20 μg/L indicate excessive exposure, while values exceeding 5–10 μg/L may be associated with neurological symptoms. In hair, which is used as a biomarker for methylmercury exposure, a concentration of approximately 1 μg/g or less is considered typical for the general population [29].

Exposure to environmental factors, including mercury, can influence BP and the cardiovascular system [11]. Chronic exposure to this metal can cause endothelial dysfunction, thrombosis, and atherosclerosis, increasing the risk of stroke and coronary heart disease. Clinical effects also include arrhythmias and kidney dysfunction [30]. Hypertension (BP) is a key risk factor for CVD, and its elevation can begin in childhood. Elevated BP in children increases the risk of future hypertension, coronary heart disease, left ventricular hypertrophy, and atherosclerosis [11]. Although subclinical vascular changes may develop early, the evidence on mercury’s impact on BP in children remains inconsistent, likely due to differences in exposure levels and co-occurring factors such as diet and measurement method [11,25]. A study conducted within the New Hampshire Birth Cohort Study found that childhood exposure to mercury was associated with increased diastolic blood pressure (DBP) and mean arterial pressure (MAP). In contrast, systolic blood pressure (SBP) showed no significant change. Boys and children with lower birth weight were more susceptible to the effects of Hg. Mechanisms of this effect may involve oxidative stress, endothelial dysfunction, and impaired kidney function, leading to increased vascular resistance and altered BP regulation [11]. Similar effects are observed with prenatal exposure to MeHg. Although the study by Costet et al. [31] did not find significant changes in SBP, DBP, or MAP, a reduced heart rate variability was observed in seven-year-old children. This is a subclinical marker of autonomic dysfunction and may serve as an early biomarker for CVD risk. The study by Hill et al. [32] found that chronic exposure to airborne Hg was associated with greater arterial stiffness and increased intima-media thickness of the arteries, suggesting a potential role in the development of atherosclerosis. Further evidence of mercury’s impact on the cardiovascular system comes from a study by Liu et al. [33], which found that boys with higher exposure to Pb and Hg had lower resting heart rates (RHRs), suggesting chronic under-arousal of the autonomic nervous system. This effect was not observed in girls, indicating possible sex differences in physiological responses to heavy metals. Proposed mechanisms include enzymatic disruption, oxidative stress, and the synergistic effects of Pb and Hg on heart function. Moreover, the study by Zhang et al. [22] found that higher levels of MeHg were significantly associated with increased total cholesterol (TC) levels, particularly in girls. This suggests that MeHg may modulate hormonal activity and influence lipid profiles, which could affect cardiovascular health later in life.

In summary, the available studies suggest a potential impact of mercury on the cardiovascular system in children, though its mechanisms and clinical significance remain unclear. The effects of exposure depend on age, sex, mercury levels, and additional factors such as diet and co-exposure to other heavy metals. Reducing prenatal exposure to methylmercury may play an essential preventive role in protecting cardiovascular health later in life. Although effects do not always manifest as elevated BP, autonomic nervous system function alterations may be early warning signs of future circulatory disturbances. Further research is necessary to understand the long-term consequences of this exposure better and to establish preventive strategies to minimize its impact on children’s health.

## 3. Lead

Lead is a chemical element with wide industrial applications, primarily occurring in the form of inorganic compounds such as lead oxide, lead nitrate, or lead acetate. It exists in three oxidation states: 0, +2, and +4, with its most common forms including metals, oxides, salts, and lead soaps. The toxicity of Pb depends on the level and duration of exposure [4,34]. Lead is present in the environment, particularly in soil and groundwater, mainly due to industrial activities and anthropogenic pollution [35]. The primary source of human lead exposure in the past was atmospheric dust containing lead, mainly from vehicle exhaust. Although air lead concentrations have significantly decreased in recent decades, blood lead levels have only dropped by a factor of 3 to 6, suggesting that internal sources of exposure, such as lead mobilization from body tissues, are playing an increasingly important role [36]. Lead, along with mercury and arsenic, is among the most toxic metals capable of crossing both the placenta and the blood–brain barrier, enabling their accumulation in fetal tissues [37]. Lead can be intensely absorbed and accumulated in bone tissue, leading to long-term persistence in the body [32]. It is estimated that in children, bones store about 75% of the total lead content, while in adults, this figure exceeds 90% [3,4,5,6,7]. This element can remain stored in bones for decades; however, aging, pregnancy, or osteoporosis may trigger its mobilization and re-release into the bloodstream [38]. Studies indicate adverse health effects when blood lead levels exceed 10 μg/dL [39]. In China, the median blood lead concentration in males aged 0 to 18 was 48.8 μg/L; in females, it was 46.1 μg/L. In contrast, in the United States, the average blood lead levels among adolescents were 22.8 μg/L in white individuals, 45.5 μg/L in Black individuals, and 40.7 μg/L in Hispanics and Mexicans [40].

Although the mechanisms of lead toxicity are not yet fully understood, studies indicate that its primary targets are enzymes involved in heme biosynthesis and antioxidant systems. Even at low blood concentrations, Pb can inhibit the activity of these enzymes, leading to increased ROS production and intensified oxidative stress, which is considered a key element of its toxicity. Excess ROS damages cells, including those of the cardiovascular system, disrupting their integrity [4,39]. Increased ROS production damages vascular endothelial cells, leading to chronic inflammation, fibrosis, and apoptosis of smooth muscle cells in the walls of resistance arteries, thereby promoting the development of hypertension [41]. In the context of chronic lead exposure, especially in children and young individuals, additional endothelial damage has been observed, which may initiate or accelerate the development of atherosclerosis. This mechanism is based on heightened inflammation and oxidative stress, worsening endothelial dysfunction and degeneration [42,43]. The study by Lee et al. [44] confirms that exposure to lead and cadmium is associated with increased levels of endothelial microparticles (EMPs) and platelet microparticles (PMPs), which are recognized as biomarkers of vascular injury and atherosclerosis progression. In the context of hypertension and atherosclerosis development, special attention is given to the effects of lead on vascular tone regulation and endothelial function. One of the key mechanisms is the reduced bioavailability of •NO, which regulates vascular tone, and the enhanced activation of pro-oxidative enzymes such as NADPH oxidase, exacerbating oxidative stress and cellular damage. Additionally, lead disrupts angiogenesis and promotes electrical dysfunction of the heart by impairing impulse conduction and prolonging action potential duration, thereby increasing the risk of arrhythmia and hypertension [14,30,41,45]. The toxic effects of lead also involve the kidneys and nervous system, especially in children who, due to the immaturity of enzymatic defense systems, are significantly more susceptible to oxidative stress and its consequences [12,13]. Prenatal lead exposure has been associated with long-term cardiovascular outcomes, including altered regulation of the hypothalamic–pituitary–adrenal axis and increased BP, particularly among children born preterm (<37 weeks) [46,47]. Early lead exposure exacerbates these processes, deepening endothelial dysfunction and disrupting vascular homeostasis [14]. Even low blood lead levels may disrupt BP and increase its value, possibly through effects on the autonomic nervous system [27]. Moreover, very low blood lead concentrations, well below the CDC’s 10 μg/dL threshold, may dysregulate this system, leading to increased vascular resistance, reduced cardiac output, and diminished stroke volume in response to stress stimuli. Lead may induce metabolic damage to tissues, vascular remodeling, and increased vascular resistance, and may also contribute to myocardial dysfunction, which, in the long term, can lead to cardiomyopathy [30,42]. It has been demonstrated that Pb^2+^ mimics calcium (Ca^2+^) and disrupts the function of ion channels, such as Cav1.2, thereby affecting vascular contractility and cardiac rhythm [45]. Studies have shown that children exposed to lead have smaller left ventricles and impaired systolic function, which may result from chronic inflammation and prolonged exposure to cardiovascular toxins [48].

Exposure to lead in children and adolescents is associated with a range of adverse hematological and metabolic changes that promote the development of CVD. In children, an increased platelet count and elevated hematocrit levels are observed, which may impair normal blood flow and accelerate atherosclerotic processes [39]. Adolescents with elevated blood lead levels have also been found to have higher LDL cholesterol levels, one of the key risk factors for heart disease [40]. Additionally, lead can activate endothelial cells and macrophages, stimulating the release of proinflammatory cytokines such as interferon gamma-induced protein 10 and calprotectin, which enhance leukocyte adhesion to the vascular wall and exacerbate inflammation [30]. Elevated blood lead levels are also accompanied by increased levels of other inflammatory markers, including IL-6 and lipoprotein-associated phospholipase A2, raising the risk of cardiovascular events [39].

Early-life exposure to lead may have long-term consequences for cardiovascular function and increase the risk of developing hypertension, atherosclerosis, and other serious CVD in the future.

## 4. Cadmium

Cadmium occurs in eight natural isotopes, but not in elemental form; instead, it is found as compounds such as cadmium oxide, carbonate, or sulfide. It is widely used in industry, but its toxicity poses a serious health risk [30]. Cadmium is a metal with no physiological function in the human body, and its presence may lead to adverse biological effects, mainly when exposure occurs during the prenatal period [37]. The primary sources of human exposure to this metal are food (primarily rice, leafy vegetables, cereal products, and shellfish), water, air, and tobacco smoke [30,49,50]. In the case of mothers exposed to cadmium, the metal can be transferred to the infant’s body through breast milk [50]. Cadmium has a strong tendency to accumulate in the body. Its biological half-life ranges from 10 to 30 years, with the kidneys and liver being the main storage sites [49,51]. Approximately 12% of Europe’s adult population exceeds cadmium’s reference levels in urine [42]. It has also been shown that urinary cadmium levels increase with age [51,52]. Even low-level exposure during childhood can lead to long-term health problems [11,42]. Cadmium levels ≥ 0.48 μg/g may be associated with increased all-cause mortality, while levels ≥0.88 μg/g are linked to a higher risk of heart attack in women [53]. Cadmium toxicity is driven by its ability to damage cellular systems, mainly through disrupting DNA repair, triggering cell death, and increasing the production of ROS [4]. Cadmium disrupts iron homeostasis, increasing oxidative stress and lipid peroxidation [51]. An increase in ROS levels promotes inflammation and vascular dysfunction. Additionally, cadmium affects the expression of antioxidant enzymes, weakening the body’s ability to neutralize oxidative stress [51]. Cadmium toxicity also involves mitochondrial dysfunction, which further enhances oxidative stress in the cardiovascular system [42]. Oxidative stress in children induced by cadmium, even at low concentrations, may increase the risk of damage to developing organs such as the heart and blood vessels, and may also contribute to cancer development later in life [35,54]. The toxic effects of cadmium involve mitochondrial dysfunction, impaired apoptosis, and oxidative stress, which can lead to genetic damage, cancer development, and tissue injury, including in the vascular system [55]. Early-life exposure to cadmium may contribute to increased cardiovascular risk through mechanisms involving oxidative stress, inflammation, and disturbances in lipid metabolism [14]. Acute cadmium exposure increases BP, whereas long-term, chronic exposure leads to its reduction, and higher environmental exposure to cadmium is associated with lower pulse wave velocity [32]. Chronic cadmium exposure in children is associated with reduced vascular elasticity and increased left ventricular mass (LVM), suggesting early structural changes in the cardiovascular system [32]. Studies conducted among children demonstrated that both prenatal and early childhood exposure to cadmium were significantly associated with increased systolic (SBP) and DBP, as well as decreased levels of TC, high-density lipoprotein (HDL), and low-density lipoprotein (LDL), indicating metabolic disturbances and early signs of cardiovascular risk [56,57]. A cohort study conducted in China showed that prenatal exposure to a mixture of heavy metals, including cadmium, was associated with changes in BP in children aged 5–6 years. However, cadmium alone did not show statistically significant associations in single-metal analyses [58].

Cadmium exposure, even at low levels during early life, can impair biological homeostasis by inducing oxidative stress, disrupting lipid metabolism, and altering vascular function. These disturbances may contribute to the early onset of cardiovascular and metabolic disorders, highlighting the need for preventive strategies, especially in vulnerable populations like children.

## 5. Arsenic

Arsenic is a widely occurring toxic metal, with contaminated groundwater being a significant source of exposure in children, especially in regions with high concentrations of this element [14]. In many Southeast Asian countries, such as Bangladesh, India, and Indonesia, arsenic levels in water and rice often exceed permissible limits, exposing millions of people to the toxic effects of this element, particularly its inorganic form [59]. In contrast, data from European countries show that exposure levels in about 40% of adolescents exceed health-based reference values, raising serious concerns about their potential impact on cardiovascular health [42]. It has also been observed that the presence of cadmium may promote the accumulation of arsenic in the heart by inducing an arsenic-binding molecule [30].

In a study conducted among approximately 200 children in Bangladesh, long-term use of drinking water with high arsenic concentrations (>50 µg/L) was associated with impaired endothelial function, as assessed by the reactive hyperemia index (RHI), indicating early subclinical vascular changes [60]. A study by Osorio-Yáñez [10] demonstrated that children aged 3–8 years who were exposed to inorganic arsenic had significantly higher systolic and DBP, increased LVM, and reduced ejection fraction (EF) and shortening fraction (SF), indicating impaired systolic heart function already in early childhood. A considerable portion of the children in the study exhibited concentric left ventricular remodeling, an early marker of CVD risk. A marginal association was also observed between urinary arsenic levels and increased left atrial diameter, suggesting the onset of structural heart changes resulting from arsenic exposure. Additionally, other studies conducted by the same research group showed a significant association between children’s exposure to arsenic and thickening of the carotid artery’s intima-media layer (cIMT), which is considered an early marker of subclinical atherosclerosis. It was also demonstrated that arsenic affects the level of asymmetric dimethylarginine (ADMA), an endogenous inhibitor of NO. The observed increase in cIMT among children from arsenic-exposed regions may therefore result both from direct toxic effects and from oxidative mechanisms associated with impaired •NO production [9]. These findings are also supported by a study conducted in the United States by Gump et al. [60], which showed that higher urinary arsenic levels in children aged 9–11 years were associated with increased cIMT and concentric hypertrophic left ventricular remodeling. Notably, this study found no association between arsenic levels and drinking water or exposure to tobacco smoke; instead, the primary exposure source was industrial urban environmental pollution, suggesting that local environmental conditions play a key role in inorganic arsenic exposure among children.

During fetal development, arsenic crosses the placenta, leading to an increased risk of miscarriage, low birth weight, fetal growth restriction, and elevated neonatal mortality [61]. Inorganic arsenic (iAs) levels in umbilical cord blood are often similar to maternal levels, indicating easy transplacental passage and significant fetal exposure during prenatal development [62]. Later in life, this element may contribute to an increased risk of CVD, cancers, and immune system disorders [61]. Moreover, recent studies indicate that arsenic may also interact synergistically with mercury. At low exposure levels, their combined effect may exacerbate the adverse impact on the children’s cardiovascular system. Prenatal exposure to arsenic and mercury, particularly during the third trimester, correlates with elevated DBP and MAP in preschool-aged children [57]. Arsenic can disrupt oxidative homeostasis by increasing ROS production and reducing the activity of antioxidant enzymes such as SOD, which promotes oxidative stress and cellular damage. Activation of the transcription factor NF-κB by iAs, along with increased expression of pro-inflammatory cytokines such as interleukin-1 beta and tumor necrosis factor alpha, may promote chronic inflammation, which plays a key role in the pathogenesis of CVD. It has also been shown that early-life exposure to iAs can impair immune system function by disrupting T and B cell responses, increasing children’s susceptibility to infections, and weakening acquired immunity [62]. Early-life exposure to this element may lead to disturbances in lipid profiles, including a decrease in HDL and an increase in atherogenic fractions such as LDL and non-HDL, as well as an unfavorable rise in the TC/HDL ratio, which is a significant risk factor for atherosclerosis [56,63].

## 6. Thallium

Thallium (Tl) is one of the most toxic metals, exhibiting higher toxicity than mercury, cadmium, arsenic, or lead. Due to its chemical properties, thallium enters the human body through ingestion, inhalation, and dermal absorption, accumulating in internal organs including the heart, liver, and kidneys. A key mechanism of its toxicity is its ionic similarity to potassium, which leads to disruption of electrolyte balance and numerous potassium-dependent enzymatic processes essential for normal physiological function. Thallium particularly affects mitochondria, triggering excessive production of ROS, which causes oxidative stress and cellular damage. Lipid peroxidation and damage to proteins and DNA caused by this oxidative stress may lead to long-term degenerative changes in the human body, especially in children, who are more vulnerable to its toxic effects [64].

Studies have shown that even relatively low concentrations can cause early pathological changes in children’s hearts in the context of thallium’s impact on the cardiovascular system. In the study by Duan et al. [65], significant cardiac dysfunction was observed in six children exposed to thallium levels ranging from 13.4 to 60.1 mg/L. Although thallium concentrations dropped below 5 mg/L after four years, myocardial function continued to deteriorate, indicating long-lasting and potentially irreversible effects of thallium on the circulatory system. This may result from disrupted ion transport in cardiac tissue, leading to arrhythmias and weakened heart function [65]. Notably, thallium is highly retained in the body and excreted slowly. Its biological half-life ranges from 10 to 30 days, and in some cases, it can be detected even years after exposure [64]. Children are particularly susceptible to thallium toxicity; according to studies, as little as one-tenth of the adult lethal dose may pose a life-threatening risk to a child [64]. Chronic exposure to this heavy metal has also been linked to damage in the liver, kidneys, and nervous system. In children exposed prenatally to thallium, increased risks of miscarriage, low birth weight, and neurological deficits have been observed. Furthermore, research indicates that thallium can cross the placental barrier and be excreted in breast milk, potentially affecting neonatal health [65]. Importantly, recent population-based research has demonstrated an inverse correlation between urinary thallium levels and the prevalence of hypertension in children and adolescents aged 8–17 years. Higher urinary thallium concentrations were statistically associated with a lower risk of hypertension, particularly among boys [66].

These findings highlight the urgent need for stricter exposure limits for thallium, especially for children, as current safety standards do not fully account for the long-term effects of low-level exposure. Further research is needed to understand thallium toxicity mechanisms and develop more effective detoxification methods [65].

**Figure 1:**
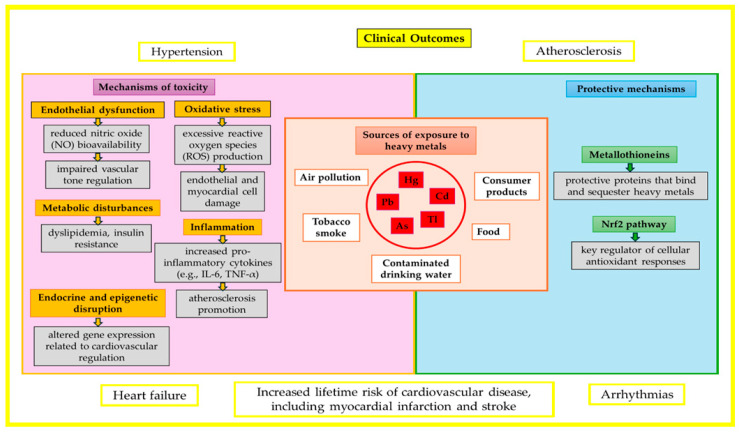
The impact of heavy metals on the cardiovascular system in children.

Figure 1 illustrates the toxicological mechanisms and clinical effects of heavy metal exposure on the cardiovascular system in children.

## 7. Sources of Heavy Metal Exposure and Diagnostic Guidelines

Children are particularly vulnerable to environmental exposure to toxic metals due to their developing physiology, higher relative intake of air, water, and food, and behaviors such as hand-to-mouth activity and close contact with soil and dust. Exposure can occur through multiple pathways, including ingestion, inhalation, and dermal absorption, with significant variation depending on regional industrial activity, socioeconomic status, and public health infrastructure. Lead is commonly found in contaminated soil, deteriorating lead-based paint in older buildings, and drinking water transported through lead pipes. Despite bans in many countries, legacy lead contamination remains a persistent threat in urban environments. Additionally, inexpensive toys, imported ceramics, metal dishes, cutlery, packaging, and household dust can serve as sources of lead exposure [67,68,69]. The FDA highlights the possibility of lead entering food during the production process. Food contaminated with lead may therefore affect children’s health both directly and indirectly through the mother [70]. Other sources of lead exposure include food products such as cereals, leafy vegetables, and potatoes. In breastfeeding women, higher levels of lead may also occur as a result of its release into the bloodstream due to bone demineralization during pregnancy and lactation [71]. Traditional cultural products, such as bronze or brass cookware [72], spices, toys, and synthetic and natural cosmetics [73,74], may also serve as unidentified sources of lead exposure. Mercury exposure in children often occurs through consuming fish containing methylmercury, huge predatory species like tuna, swordfish, or shark, and shellfish. Other sources include mercury-based dental amalgams, skin-lightening creams, certain vaccines preserved with thimerosal, and vapors released from broken thermometers or fluorescent bulbs [75,76,77,78,79,80,81,82,83]. Attention is also drawn to mercury contamination of food products such as herbs, spices, dietary supplements, meat, cereal products, mushrooms, vegetables, and fruits, as well as oils, fats, and medicines [82]. Cadmium is found in contaminated food and water, particularly rice, leafy vegetables, and shellfish. It is also present in cigarette smoke, including secondhand smoke, making children of smoking caregivers particularly susceptible. Industrial emissions and using phosphate fertilizers further contribute to cadmium contamination in air and soil [83,84,85]. Arsenic exposure is widespread in regions with contaminated groundwater, where it may be present in drinking water at concentrations far exceeding recommended safety levels. Arsenic is also found in rice, especially when irrigated with arsenic-laden water, as well as in some pesticides and wood preservatives [73,86,87,88]. Thallium, though less frequently discussed, is highly toxic and may be present in areas with mining or industrial activity. It can enter the body through contaminated water, food, air, or direct skin contact. Thallium can also be present in rodenticides or electronic waste, posing risks to children living near disposal or recycling sites [89,90].

In addition to these specific sources, the indoor environment plays a significant role. Children may be exposed to toxic metals from household dust, painted surfaces, cosmetics, or herbal remedies, particularly in regions lacking regulatory oversight. Despite Regulation (EC) No 1223/2009 of the European Union, banning the use of heavy metals in cosmetics, their presence is sometimes tolerated if it is technically unavoidable. Costume cosmetics, especially face paints used by children, may contain toxic metals such as lead, cadmium, and arsenic, sometimes exceeding safety limits. Absorption through the skin or accidental ingestion poses a potential health risk, particularly with frequent or prolonged use [91]. Socioeconomic disparities often compound these risks, as low-income communities are likelier to live near industrial areas or in poorly maintained housing with legacy contaminants.

Understanding these environmental sources is critical to designing effective public health interventions and policies to reduce exposure in pediatric populations. The diagnosis of heavy metal exposure in children, including prenatal exposure, presents numerous analytical challenges (related to the selection of appropriate biological matrices and sample collection) as well as clinical challenges (concerning the interpretation of results in the context of symptoms and exposure history). Depending on the type of metal, timing, and route of exposure, various biological matrices such as blood, urine, hair, and nails may be used [92]. In prenatal exposure assessment, maternal blood and umbilical cord blood are primarily utilized. In biomonitoring studies, the placenta may also serve as an alternative matrix [93]. Guidelines for the diagnostic evaluation of heavy metal exposure, including recommendations on screening in children, are outlined in the document Heavy Metals in Baby Foods and Fruit Juices. Advice for Families from Environmental Pediatricians, prepared by the New York State Children’s Environmental Health Centers in collaboration with the American Academy of Pediatrics, recommends routine blood lead screening at 12 and 24 months of age and lead exposure risk assessment for all children aged 6 months to 6 years. It also states that testing for other metals, such as arsenic, mercury, and cadmium, should only be considered when there is documented environmental risk. The guidelines strongly emphasize preventive measures and minimizing children’s contact with potential sources of heavy metals in their environment [94].

## 8. Prenatal Exposure to Heavy Metals and the Risk of Congenital Heart Defects

Growing evidence indicates that prenatal maternal exposure to toxic substances, including heavy metals, plays a significant role in increasing the risk of congenital disabilities in offspring [95]. Table 1 presents selected mechanisms of the toxic effects of heavy metals and their impact on the cardiovascular system in children, based on available epidemiological studies. However, none of the analyzed studies include data on the acute effects of heavy metal exposure or compare symptoms resulting from acute versus chronic exposure. Nevertheless, the reviewed literature provides consistent evidence that chronic, low-level exposure to heavy metals, particularly arsenic, is associated with subtle alterations in cardiovascular function and structure in children, even in the absence of overt clinical symptoms. These alterations include elevated systolic and diastolic blood pressure, reduced EF and SF, and increased aortic root diameter (ARD) and left atrial diameter (LAD) [9,10,11,58].

Congenital heart defects (CHDs) are among the most common developmental anomalies and are responsible for a significant proportion of infant mortality worldwide. Their etiology is complex and includes both genetic and environmental factors, such as air pollution and heavy metals. Many cases are thought to result from the interaction of multiple contributing factors [96]. Fetal heart development is a multistage process dependent on the interplay of genetic and environmental factors, including exposure to teratogenic substances. Particular importance in the etiology of CHDs is attributed to disturbances in the migration and differentiation of cells derived from the heart field and neural crest, as well as disruptions in signaling pathways essential for forming the atrial and ventricular septa and the great vessels [97]. Defects such as ventricular septal defect, atrial septal defect, transposition of the great arteries, and patent ductus arteriosus are among the most common congenital heart defects and are associated with abnormal development of cardiac structures, including septation and formation of the great vessels during early embryogenesis [97,98,99,100]. Among these, transposition of the great arteries exemplifies a common defect of the conus and arterial trunk, alongside tetralogy of Fallot and common arterial trunk [97]. The Wnt, BMP, and Notch signaling pathways play essential roles in cardiac embryogenesis. The Wnt pathway is involved in progenitor cell proliferation and the septation of the outflow tract and atrioventricular region [101]. The BMP pathway regulates the formation of endocardial cushions, cardiac septation, and the development of the great vessels [102]. The Notch pathway influences the endocardial-to-mesenchymal transition, valve development, and the formation of the interventricular septum [103]. Maternal exposure to higher levels of lead, arsenic, cadmium, and mercury is associated with an increased risk of severe forms of CHDs in offspring [104]. These metals may interfere with the development of cardiac structures by inducing oxidative stress, disrupting calcium homeostasis, damaging genetic material, and affecting signaling pathways critical to embryogenesis, such as Wnt, BMP, and Notch, as well as by impairing placental function [101]. In addition, these metals may lead to epigenetic modifications, such as DNA methylation and histone acetylation, which disrupt the regulation of genes involved in cardiovascular development [105,106]. Placental dysfunction caused by heavy metals may further impair the transport of oxygen and micronutrients, increasing the risk of embryonic hypoxia and its teratogenic consequences [104]. These observations are consistent with the so-called Barker hypothesis, which suggests that environmental stress during fetal life increases the risk of cardiovascular diseases in adulthood [96]. Table 2 presents heavy metals most frequently described in the literature as potential teratogens associated with the occurrence of congenital heart defects.

## 9. Preventive Strategies and Public Health Implications

Given the widespread presence of toxic metals in the environment and their proven harmful effects on the cardiovascular system in children, preventive strategies are essential. Effective interventions should focus on minimizing exposure, especially during critical developmental periods. Regarding policy and regulatory strategy, it is advisable to strengthen environmental protection regulations to reduce industrial emissions of lead, cadmium, and mercury and to introduce bans on the use of hazardous substances in toys and consumer products. In terms of monitoring and screening, regular biomonitoring of heavy metal levels in at-risk populations, especially in children living near industrial or mining areas, should be implemented. Another aspect is educating parents and caregivers about sources of exposure, e.g., avoiding fish with high mercury content, such as swordfish or tuna, in the diet of young children, or limiting the use of cosmetics and traditional medicines containing mercury or lead. In the case of nutritional interventions, promoting diets rich in iron, calcium, zinc, and antioxidants, which can reduce metal absorption and counteract oxidative stress, may be effective. Providing access to clean drinking water is equally, if not the most, important, especially in regions where arsenic contamination is endemic. Sustainable urban planning using remediation of contaminated soils and implementing green zones around industrial areas is also a valuable strategy. Together, these actions can significantly reduce the burden of toxic metal exposure in children and improve cardiovascular and general health outcomes.

## 10. Conclusions

Heavy metals such as mercury, lead, cadmium, arsenic, and thallium exhibit strong toxic effects, particularly in children, who are more vulnerable to their impact. Mercury, especially in methylmercury, accumulates in organs and may induce oxidative stress, endothelial dysfunction, and cardiovascular disturbances. Lead damages vascular cells, disrupts cardiac conduction, and can contribute to atherosclerosis and hypertension, even at low exposure levels. Cadmium disrupts oxidative balance, damages genetic material, affects endothelial and cardiac function, and may lead to metabolic disorders. Arsenic, particularly in its inorganic form, can harm the cardiovascular system as early as childhood, crosses the placental barrier, and may trigger epigenetic changes that increase the risk of CVD. Thallium, although less commonly discussed, is highly toxic, accumulates in the body, and may cause permanent heart damage in children. All these metals exert their effects through mechanisms involving oxidative stress, inflammation, and metabolic disruption, with health consequences that may emerge early in life and persist long-term. Therefore, beyond identifying toxic effects, emphasis should be placed on comprehensive preventive measures involving environmental regulation, public health education, and nutritional strategies to protect vulnerable pediatric populations.

## Figures and Tables

**Table 1:** Cardiovascular effects of heavy metal exposure in children.

Metal	Clinical Outcomes	Proposed Mechanism	Study Population	Ref.
Mercury	↓ HR in boys; no effect in girls	Autonomic nervous system dysfunction affecting HR, partly due to oxidative stress Combined myocardial and autonomic disruption via oxidative stress (observed in co-exposure to Hg and Pb)	532 adolescents; mean age 12 years	[33]
	↑ cIMT, and PWV; no effect on HR or BP	Endothelial dysfunction, partly due to oxidative stress	291 children, aged 9–11 years	[32]
	↑ DBP, MAP; no effect on SBP		395 children, aged 5–6 years	[11]
	↑ DBP, MAP at age 5–6, esp. 3rd trimester		2534 children	[57]
	↑ TC (notably in girls); no effect on SBP or DBP	Possible lipid metabolism disruption indicates potential early CVD risk	1129 adolescents, aged 12–19 years	[22]
Lead	Slight ↑ HR in boys; no effect in girls	Autonomic nervous system dysfunction affecting HR, partly due to oxidative stress	532 adolescents, mean age 12 years	[33]
	↑ SBP in preterm children (<37 GA weeks), with potential long-term cardiovascular risk	Combined effects of impaired nephrogenesis, oxidative stress, and endothelial dysfunction may contribute to long-term cardiovascular risk	565 children, aged 4–6 years	[47]
	↓ LVPW, LVM, EF, SF	Oxidative stress-induced myocardial remodeling and inflammation	486 children, ages 2–6 years	[48]
	↑ LDL; no effect on SBP or DBP	Possible lipid metabolism disruption indicates potential early CVD risk	11,662 adolescents	[40]
	None confirmed in either study	No cardiovascular effects observed	291 children, aged 9–11 years	[32]
			2534 children	[57]
Cadmium	↓ SBP, DBP, HR; ↑ LVM; ↓ PWV	Vascular and autonomic dysfunction	291 children, aged 9–11 years	[32]
	↓ TC, HDL, LDL; ↑ SBP, DBP	Altered lipid metabolism with unclear impact on cardiovascular risk	540 children, ages 4.5 and 9 years	[56]
	No cardiovascular effects observed	None confirmed in either study	2534 children	[57]
Arsenic	↓ TC, HDL	Altered lipid metabolism with unclear impact on cardiovascular risk	540 children, ages 4.5 and 9 years	[56]
	↑ DBP, SBP, MAP at age 5–6, esp. 3rd trimester	Endothelial dysfunction, partly due to oxidative stress	2534 children	[57]
	↑ SBP, DBP, LVM, LAD (marginal); ↓ EF, SF	Endothelial dysfunction mediated by oxidative stress and vascular remodeling	161 children, ages 3–8 years	[10]
	↑ cIMT; no effect on lipids	Endothelial dysfunction mediated by ADMA	199 children, ages 3–14 years	[9]
	↑ cIMT, concentric cardiac hypertrophy	Early cardiovascular alterations due to endothelial dysfunction	245 children, ages 9–11 years	[60]
	↑ LDL, TC, non-HDL, TC/HDL ratio	Widespread dyslipidemia is associated with early cardiovascular risk	237 children, aged approximately 2, 5, 8, 11, and 14 years	[63]
Thallium	↓ LDL, TC; ↑ phosphocreatine kinase, creatine kinase isoenzyme; ↓ ischemia-modified albumin	Altered lipid metabolism with unclear impact on cardiovascular risk Subclinical myocardial injury contributes to increased cardiovascular risk	6 children	[65]
	↓ prevalence of hypertension with higher urinary Tl	No clear mechanism	2295 children, ages 8–17 years	[66]

Asymmetric dimethylarginine (ADMA); Blood pressure (BP); Carotid intima-media thickness (cIMT); Diastolic blood pressure (DBP); Ejection fraction (EF); Gestational age (GA); High-density lipoprotein (HDL); Heart rate (HR); Left atrial diameter (LAD); Left ventricular posterior wall (LVPW); Left ventricular mass (LVM); Low-density lipoprotein (LDL); Pulse wave velocity (PWV); Mean arterial pressure (MAP); Shortening fraction (SF); Systolic blood pressure (SBP); Total cholesterol (TC); Total cholesterol to high-density lipoprotein ratio (TC/HDL).

**Table 2:** Types of congenital heart defects associated with heavy metal exposure.

Category	Types of CHDs	Heavy Metal	Potential Effects on the Developing Heart	Ref.
Septal defects	Atrial septal defect	Mercury	Oxidative stress, epigenetic disruption	[104]
		Arsenic		[107]
	Ventricular septal defect	Mercury		[104]
	Atrioventricular septal defect	Mercury		[104]
		Arsenic		[107]
Conotruncal defects	Tetralogy of Fallot	Mercury		[104]
	d-transposition of the great arteries	Mercury		[104]
	Truncus arteriosus	Mercury		[104]
Isolated defects	Patent ductus arteriosus	Arsenic		[107]
CHDs (general)	-	Lead		[108]

CHDs—congenital heart defects.

## Abbreviations

The following abbreviations are used in this manuscript:

ADMA	Asymmetric dimethylarginine
BP	Blood pressure
BMP	Signaling pathway
cIMT	Carotid intima-media thickness
CAT	Catalase
CHDs	Congenital heart defects
CRP	C-reactive protein
CVD	Cardiovascular disease
DBP	Diastolic blood pressure
EF	Ejection fraction
GA	Gestational age
GPx	Glutathione peroxidase
GSH	Glutathione
HR	Heart rate
HDL	High-density lipoprotein
IL-6	Interleukin-6
iAs	Inorganic arsenic
LAD	Left atrial diameter
LDL	Low-density lipoprotein
LVM	Left ventricular mass
MAP	Mean arterial pressure
•NO	Nitric oxide radical
Notch	Signaling pathway
PWV	Pulse wave velocity
ROS	Reactive oxygen species
RHR	Resting heart rate
SBP	Systolic blood pressure
SF	Shortening fraction
SOD	Superoxide dismutase
TC	Total cholesterol
TC/HDL	Total cholesterol to high-density lipoprotein ratio
TNF-α	Tumor necrosis factor alpha
Wnt	Signaling pathway
